# Evaluation of Lymphocyte-to-Monocyte Ratio and Mean Platelet Volume-to-Platelet Ratio in Rhegmatogenous Retinal Detachment

**DOI:** 10.1155/2022/9127745

**Published:** 2022-09-16

**Authors:** Xuan Xiao, Jiayi Song, Lin Ye, Wen Zuo, Hongxia Yang, Changzheng Chen, Hongmei Zheng, Ting Chen

**Affiliations:** ^1^Department of Ophthalmology, Renmin Hospital of Wuhan University, Wuhan, Hubei, China; ^2^Department of Eye Plastic and Lacrimal Diseases, Shenzhen Eye Hospital, Shenzhen, China

## Abstract

**Purpose:**

To assess the predictive value of inflammatory blood biomarkers in rhegmatogenous retinal detachment (RRD) patients and its correlation with proliferative retinopathy (PVR) grade.

**Methods:**

82 RRD patients and 1 : 1 age- and gender-randomly matched cataract patients as the control group were included. The clinical features and laboratory parameters of all participants were collected and recorded, and the comprehensive index of inflammatory blood and its correlation with PVR were calculated.

**Results:**

Monocytes and monocyte-to-high-density lipoprotein ratio (MHR) were significantly lower (*P*=0.005, *P*=0.044), while mean platelet volume (MPV), lymphocyte-to-monocyte ratio (LMR), and MPV-to-platelet ratio (MPR) were significantly higher in RRD patients as compared with the control group (*P*=0.013, *P*=0.019, *P*=0.037). LMR and MPR might be the predictors of RRD. The receiver operating characteristics analysis showed that the values of MPV, LMR, and MPR in RRD patients were 0.612, 0.606, and 0.594, respectively. PVR grade was not associated with inflammatory blood biomarkers.

**Conclusion:**

The increase in MPV, LMR, and MPR were associated with increased odds of RRD. LMR and MPR may be useful as inexpensive and effortless biomarkers for assessing the occurrence of RRD.

## 1. Introduction

Rhegmatogenous retinal detachment (RRD) is a vision-threatening disease requiring urgent intervention. It is characterized by the separation of the neurosensory retina from the underlying retinal pigment epithelium (RPE) during a retinal break. The reported annual incidence of RRD is 1/10 000 population [1]. The increased risks of RRD are increased age, previous cataract surgery, myopia, and trauma [2]. In addition to the structural changes in the retina, complex biomolecular mechanisms activated after RRD may also play an important role in the pathogenesis. During RRD, proinflammatory and growth factors are released into the vitreous, which plays a crucial role in the injury-induced wound-healing process and apoptosis of retinal photoreceptors [3–5]. During the early repair phase of RRD, phagocytic cells such as neutrophils, monocytes, macrophages, and platelets participate in the immune reactions [4, 6]. Proliferative vitreoretinopathy (PVR), a pathological wound-healing process of cellular proliferation and contraction, is the most common cause of failure of reattachment surgery of RRD [7, 8]. In PVR, an inflammatory and scarring response may also develop [9, 10].

Changes in the number and composition of complete blood count (CBC) are reliable indicators of systemic inflammation. As the inexpensive and widely available features, the neutrophil-to-lymphocyte ratio (NLR), platelet-to-lymphocyte ratio (PLR), lymphocyte-to-monocyte ratio (LMR), mean platelet volume-to-platelet ratio (MPR), and monocyte-to-high-density lipoprotein ratio (MHR) have been investigated as predictors of progression and prognostic biomarkers in several diseases [11–15]. Those markers have also been investigated and indicate their potential role in ocular diseases such as central retinal artery occlusion, retinal vein occlusion, and glaucoma [16–21]. To our best knowledge, the roles of NLR, PLR, LMR, MPR, and MHR in RRD have not been detected yet, especially whether the above biomarkers are associated with PVR. Hence, the purpose of this study was to evaluate the inflammatory blood biomarkers in patients with RRD and explore their relationship with PVR in the clinical setting of RRD.

## 2. Materials and Methods

This retrospective case-control study was approved by the Ethical Committee Board of Renmin Hospital of Wuhan University and followed the tenets of the Declaration of Helsinki. The digital medical records of RRD patients or cataract patients who were diagnosed and under surgery in the Department of Ophthalmology, Renmin Hospital of Wuhan University, Wuhan, China, between January 2020 and December 2020 were analyzed.

The cataract patients were randomly chosen as 1 : 1 age- and gender-matched controls for comparisons, with no history of ocular disease (except for refractive error and cataract). The exclusion criteria were as follows: (1) systemic diseases including diabetes mellitus, hypertension, (2) autoimmune diseases, (3) renal failure, liver diseases, cardiovascular disease, chronic obstructive pulmonary disease, (4) acute or chronic infection, (5) malignancy, (6) smoking or alcohol consumption, (7) drugs used, such as steroid, nonsteroidal anti-inflammatory drugs, anticoagulant medications, and oral contraceptives, (8) history of ocular trauma, (9) history of any surgery within 3 months, (10) without the data of blood samples, and (11) age less than 18 years old. Moreover, RRD patients who had a history of ocular pathology other than refractive error/cataract/RRD or had intraocular surgery were also not included in the study.

Within 2 days of admission, all patients received a comprehensive ocular examination, including the preoperative best-corrected visual acuity (BCVA), slit-lamp examination, intraocular pressure measurement, dilated fundus examination, ocular B-scan ultrasonography, and wide-field fundus photography. BCVA was converted into the logarithm of the minimum angle of resolution (logMAR) for statistical analysis. Further, the preoperative degree of proliferative vitreoretinopathy (PVR) in RRD patients was graded according to wide-angle photography (JYS and WZ, independently). Any disagreements were determined by a more senior physician (TC). Age, gender, duration of RRD (days), history of surgery or trauma, medical condition, drug usage, and the status of smoking or drinking were also recorded. As a routine part of surgery practice, peripheral blood samples were collected from all patients on the morning of the second day of admission. The counts of white blood cells (WBC), neutrophils, lymphocytes, monocytes, platelets, and mean platelet volume (MPV) were measured. NLR, PLR, LMR, and MHR were also calculated.

### 2.1. Statistical Analysis

Quantitative data were described as mean ± SD, and compared between the RRD group and the control group with the *t*-test or the Mann–Whitney *U* test. Qualitative variables were reported as numbers and percentages and compared by *χ*^2^ test. The Spearman's correlations were used to detect the associations between the significantly correlated parameters and main clinical features. A receiving operating characteristic (ROC) curve was performed to calculate the sensitivity and specificity of the significant blood indices and the optimal cutoff value for predicting RRD. The optimal cutoff for sensitivity and specifically was calculated according to the maximal Youden Index. The areas under the ROC (AUC) curves were used to demonstrate the predictive validities. A binary logistic regression analysis was used to evaluate the association between the combined inflammatory blood biomarkers (NLR, PLR, LMR, MPR, MHR) and RRD. Results were summarized as odds ratios (OR) and 95% confidence intervals (CI). All statistical analyses were performed with IBM SPSS Statistics (version 25.0). *P* value (two-side) < 0.05 was considered statistical significance.

## 3. Results

A total of 82 RRD patients and 82 age- and gender-matched randomly selected healthy cataract patients as a control group were eligible for this study. The clinical features and laboratory parameters of these people are shown in Table 1. The mean age of the RRD group and the control group were 51.8 ± 11.2 years and 52.9 ± 10.6 years, respectively. No significant difference was found with respect to age (*P*=0.493) and gender (*P*=1.0) between the two groups. The prevalence of high myopia was similar between the RRD group and the control group (*P*=0.575). Monocytes and MHR were significantly lower in the RRD group compared to the control group (*P*=0.005, *P*=0.044), while MPV, LMR, and MPR were significantly higher in RRD patients as compared to the control group (*P*=0.013, *P*=0.019 and *P*=0.037, respectively). On the other hand, there were no statistical differences between the two groups in terms of WBC, neutrophils, lymphocytes, NLR, and PLR (*P* > 0.05 for all comparisons; Table 1).

**Figure 1:**
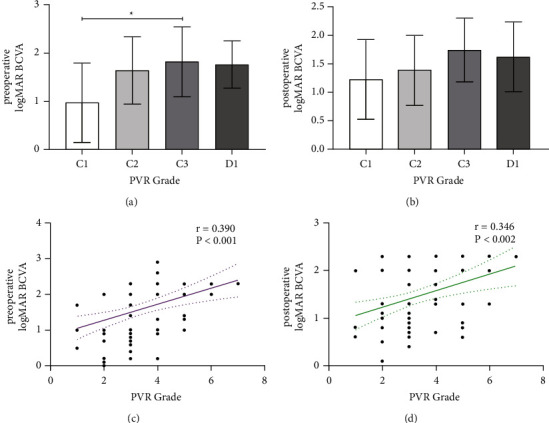
(a) Comparison of preoperative logMAR BCVA between grade C1, C2, C3, and D1 in RRD patients was statistically significant (*P*=0.023). (b) Comparison of postoperative logMAR BCVA between grade C1, C2, C3, and D1 in RRD patients was not statistically significant (*P*=0.145). (c) Correlation between preoperative logMAR BCVA and PVR grade in RRD patients (*P* < 0.001, *r* = 0.390). (d) Correlation between postoperative logMAR BCVA and PVR grade in RRD patients (*P*=0.002, *r* = 0.346). Abbreviations: logMAR, logarithm of the minimum angle of resolution; BCVA, best-corrected visual acuity; RRD, rhegmatogenous retinal detachment; PVR, proliferative vitreoretinopathy.

The PVR grading of RRD patients was as follows: grade B, 4 eyes; grade C1, 12 eyes; grade C2, 35 eyes; grade C3, 14 eyes; grade D1, 13 eyes; grade D2, 3 eyes; and 1 eye in D3, respectively. Further, the preoperative logMAR BCVA of RRD patients in grades C1, C2, C3, and D1 were compared, and the comparison of preoperative logMAR BCVA between the 4 groups was statistically significant (*P*=0.023; Figure 1). The logMAR BCVA trend of the above 4 groups after operation was similar to that of preoperative logMAR BCVA, but the difference between the grades was not statistically significant (*P*=0.145; Figure 1). Correlation analysis showed that PVR grade was significantly positively correlated with preoperative logMAR BCVA (*r* = 0.390, *P* < 0.001), postoperative logMAR BCVA (*r* = 0.346, *P*=0.002) (Figure 1), duration of RRD (*r* = 0.361, *P*=0.001), and postoperative intraocular pressure increase (*r* = 0.381, *P* < 0.001), but was not correlated with CBC parameters.

In addition to PVR grade, postoperative logMAR BCVA was significantly positively correlated with MCV (*r* = 0.304, *P*=0.007) and duration of RRD (*r* = 0.242, *P*=0.041) (Table 2). Univariate regression analysis showed that postoperative logMAR BCVA was significantly associated with age, high myopia, preoperative logMAR BCVA, and PVR grade. Then stepwise multivariate regression analysis was used to evaluate factors associated with postoperative logMAR BCVA. It was found that high myopia and PVR grade most affected postoperative visual acuity recovery (*P* < 0.001, *R*^2^ = 0.294).

High myopia, NLR, PLR, LMR, MPR, and MHR were included as covariates for binary logistic regression analysis to identify the variables associated with the diagnosis of RRD. Ultimately, LMR (OR = 1.545, 95%CI = 1.086–2.200, *P*=0.016) and MPR (OR = 1.014, 95%CI = 44.837–2.294, *P*=0.025) are related to the diagnosis of RRD.

**Figure 2:**
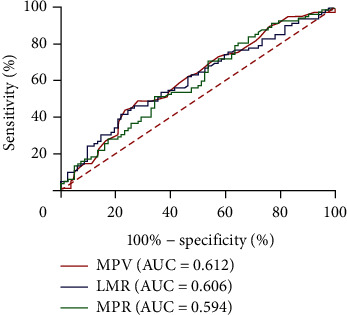
The diagnostic value of MPV, LMR, and MPR for RRD. Abbreviations: MPV, mean platelet volume; LMR, lymphocyte-to-monocyte ratio; MPR, MPV-to-platelet ratio; RRD, rhegmatogenous retinal detachment.

Considering that certain inflammatory blood biomarkers were significantly different between the RRD group and healthy controls and some of them were independently related to the diagnosis of RRD. Therefore, ROC curve analysis was further performed to evaluate the predictive value of inflammatory blood markers in the occurrence of RRD. ROC curve analysis showed that the AUC of MPV, LMR, and MPR in RRD patients were 0.612, 0.606, and 0.594, respectively (Figure 2). The best cutoff value of MPV was 11.050, with a sensitivity of 48.8% and a specificity of 72.0%. The best cutoff value of LMR was 4.752, with a sensitivity of 46.3% and a specificity of 73.2%. The best cutoff value of MPR was 0.0463, with a sensitivity of 70.7% and a specificity of 46.3%.

## 4. Discussion

The specific pathogenesis of RRD is not yet clear. Although current surgical treatments can reattach the detached retina anatomically, postoperative visual function recovery may vary greatly. In this study, the inflammatory blood biomarkers in RRD patients were analyzed for the first time. The results showed that monocytes and MHR decreased, and MPV, LMR, and MPR increased in RRD patients. The increase in MPV, LMR, and MPR was associated with increased odds of RRD. LMR and MPR might be the predictors of RRD. Postoperative logMAR BCVA was correlated with MCV, PVR grade, and duration of RRD. However, PVR grade was not associated with inflammatory blood biomarkers.

The pathological phases of RRD can be divided into the acute phase and the chronic phase, in which the PVR is the dominant characteristic. In the normal eye, no cellular junctions exist between the outer segments (OS) of the photoreceptors and the RPE, while they are adherent by the microvilli on the surface of the RPE [22]. Upon retinal detachment, the OS and RPE immediately develop ischemic and structural changes, and afterwards, the blood-retinal barrier (BRB) in the inner retina is broken down [23]. The junctions between OS and RPE are damaged and a space between these two structures is formed [22]. During the early repair period, inflammatory cells such as neutrophils, monocytes, and macrophages migrated to the damaged subretinal space [4]. Platelets were then involved in immune responses and the coagulation cascade was activated, resulting in the formation of mature scar tissue (PVR).

Recently, increasing research has estimated the common, effective, reproducible, and inexpensive blood inflammation indices in peripheral blood to find out the predictive and prognostic biomarkers in different diseases [16–18]. The levels of these markers were also found to be different between acute and chronic conditions [19, 24]. Among these inflammatory cells, monocytes are the primary cells mediating the cytokine storm following RRD [25]. At the same time, the RPE cells act as a gateway for monocyte trafficking to the retina [26, 27]. As a result of the damage of BRB in RRD, inflammatory cells such as monocytes in peripheral blood may enter the retina, resulting in the changes of these inflammatory cells and their combined indicators, as suspected in diabetic retinopathy [20]. Consistent with the assumption, significantly decreased monocyte levels were found in RRD patients in this study. The monocyte level was also positively associated with the duration of RRD and postoperative intraocular pressure increase. MPV is an important marker of platelet size and activity. Previous researchers have found increased MPV values in several thrombotic diseases [28, 29]. Platelets also participate in the immune response in various conditions [6]. During the RRD process, the activation of platelets was also investigated [25]. Platelet degranulation, blood coagulation, and other related proteins have been detected in the vitreous of RRD patients and increased with the progression of RRD, suggesting the importance of platelet activation in the process of RRD [20]. The results partially supported these findings; they found that the MPV of RRD patients increased significantly, showing an increase in blood board activity. However, the correlation between MPV and PVR grade and the duration of RRD was not found.

Lymphocytes perform the function of adaptive immunity by stratifying into *T* helper cells. Monocytes play a role in secreting proinflammatory and prooxidant cytokines [30]. Rather than individual blood inflammatory markers, which may be influenced by various physiological, pathological, and physical factors, the combination of two markers seems to have more predictive and stable values [31]. LMR is calculated by the ratio of lymphocytes to monocytes in peripheral blood. In this study, LMR was a risk factor for RRD. Simsek and Ozdal [32] also reported an increased LMR in FUS patients. Li et al. [33] showed that LMR was closely associated with primary angle closure glaucoma. On the other hand, Zhang et al. [34] found LMR significantly lower in neovascular glaucoma patients. According to these results, LMR might be used as a predictor marker for identifying the risk of RRD. For further analysis, there was no correlation between LMR and logMAR visual acuity or duration of RRD, indicating that the association between LMR and RRD was stable.

Platelets play an important role in the maintenance of vessel integrity, innate immunity, and inflammatory response and can trigger inflammation by releasing various cytokines and adhesion molecules [35]. Elevated MPV usually represents a chronic inflammatory state [36], and can also be increased in conditions such as smoking, hypertension, and dyslipidemia. MPR is the ratio of MPV to platelets, and a higher MPR is an indicator of the proinflammatory state. Akca et al. [36] found that increased preoperative MPR may indicate poor wound healing. Garcia-Pachon et al. [37] found that MPR could provide prognostic information for 1-year mortality in patients with cardiac pleural effusion due to heart failure. We found that MPR was significantly increased in RRD patients, suggesting that RRD patients may have systemic chronic inflammation. The results showed that MPR is a risk factor for RRD, but further analysis did not find a correlation between MPR and preoperative PVR grade and visual prognosis. This might be related to the fact that visual acuity and intraocular pressure were recorded for only one week after surgery, and a longer follow-up is required.

NLR and/or PLR, the common markers of systemic inflammation, have been evaluated in various diseases [18, 29, 34]. In this study, the predictive values of NLR or PLR in RRD patients were not found. As mentioned above, we analyzed the correlation between the above biomarkers and the PVR grade and found no correlation between the PVR grade and the above biomarkers, which may be related to the uneven sample size of each grade.

There are some limitations to this study. First, as a retrospective case-control study in single center limits the representativeness of the results. Second, as a retrospective study, the inflammatory cytokines in the vitreous sample could not be obtained and detected. If these cytokines in the vitreous can be detected, the correlation between the inflammatory blood markers and the vitreous in RRD patients can be analyzed. Third, a wide duration of RRD was included in this study, which may have an effect on the inflammatory status of the RRD process.

In conclusion, the increased MPV, LMR, and MPR were associated with increased odds of RRD. Despite the limitations of this study, it provided a clue that LMR and MPR may be useful as inexpensive and effortless biomarkers for assessing the occurrence of RRD. In addition, the predictive value of inflammatory blood markers for postoperative visual function recovery in RRD patients with different PVR grades needs further studies with a larger sample size. It is expected to reorient the management of surgical strategies to optimize postoperative outcomes.

## Figures and Tables

**Table 1:** Clinical features and laboratory parameters of included patients.

	RRD groups (*n* = 83)	Control group (*n* = 83)	*P* value
Age (years)	51.8 ± 11.2	52.9 ± 10.6	0.493
Gender (male/female)	45 (54.9%): 37 (45.1%)	45 (54.9%): 37 (45.1%)	1.00
High myopia (yes/no)	20 (24.4%): 62 (75.6%)	17 (20.7%): 65 (79.3%)	0.575
White blood cell (× 10^9^/L)	5.86 ± 1.53	6.33 ± 1.75	0.138
Neutrophil (× 10^9^/L)	3.37 ± 1.25	3.64 ± 1.38	0.186
Lymphocyte (× 10^9^/L)	1.91 ± 0.53	2.00 ± 0.65	0.709
Monocyte (× 10^9^/L)	0.42 ± 0.12	0.49 ± 0.15	**0.005**
Platelet (× 10^9^/L)	212.68 ± 55.94	232.90 ± 69.12	0.069
MPV (fL)	11.12 ± 1.15	10.70 ± 1.11	**0.013**
HDL-Ch (mmol/L)	1.16 ± 0.25	1.17 ± 0.28	0.893
NLR	0.56 ± 0.10	0.57 ± 0.09	0.805
PLR	119.19 ± 45.13	125.26 ± 48.43	0.254
LMR	4.83 ± 1.52	4.27 ± 1.27	**0.019**
MPR	0.06 ± 0.02	0.05 ± 0.02	**0.037**
MHR	0.39 ± 0.16	0.45 ± 0.18	**0.044**

MPV, mean platelet volume; HDL-Ch, high-density lipoprotein cholesterol; NLR, neutrophil-to-lymphocyte ratio; PLR, platelet-to-lymphocyte ratio; LMR, lymphocyte-to-monocyte ratio; MPR, MPV-to-platelet ratio; MHR, monocyte-to-high-density lipoprotein. *P* values in bold are significant.

**Table 2:** Spearman correlation between monocytes, MCV, HDL-Ch, MHR, LMR, and main clinical features in RRD patients.

Ocular parameters	Monocytes (*P* value)	MCV (*P* value)	HDL-Ch (*P* value)	MHR (*P* value)	LMR (*P* value)	PVR grade (*P* value)	High myopia (*P* value)
Age	0.053 (0.637)	**0.239 (0.031)**	0.066 (0.558)	0.005 (0.961)	−0.079 (0.481)	**0.225 (0.042)**	−**0.442 (<0.001)**
Duration of RRD	**0.259 (0.025)**	0.078 (0.507)	−0.025 (0.830)	0.177 (0.128)	−0.063 (0.589)	**0.361 (0.001)**	−**0.296 (0.010)**
High myopia	−0.065 (0.559)	−0.173 (0.119)	−0.129 (0.248)	0.022 (0.843)	0.054 (0.630)	−0.064 (0.567)	—
PVR grade	0.162 (0.147)	0.017 (0.879)	0.038 (0.732)	0.091 (0.417)	−0.060 (0.589)	—	−0.064 (0.567)
IOP	0.131 (0.249)	−0.080 (0.482)	−**0.235 (0.036)**	0.184 (0.102)	0.002 (0.984)	0.141 (0.213)	0.063 (0.579)
IIOP	**0.221 (0.049)**	0.059 (0.603))	−0.200 (0.075)	**0.266 (0.017)**	0.007 (0.950)	**0.381 (<0.001)**	0.189 (0.094)
Pre-logMAR BCVA	0.114 (0.312)	0.009 (0.933)	0.013 (0.909)	0.080 (0.476)	−0.207 (0.064)	**0.390 (<0.001)**	0.084 (0.459)
Post-logMAR BCVA	0.161 (0.156)	−**0.304 (0.007)**	0.186 (0.101)	0.016 (0.888)	−0.076 (0.507)	**0.346 (0.002)**	−**0.368 (0.001)**

MCV, mean corpuscular volume; HDL-Ch, high-density lipoprotein cholesterol; MHR, monocyte-to-high-density lipoprotein; LMR, lymphocyte-to-monocyte ratio; RRD, rhegmatogenous retinal detachment; PVR, proliferative vitreoretinopathy; IOP, intraocular pressure; IIOP, increased intraocular pressure; BCVA, best-corrected visual acuity; logMAR, logarithm of minimal angle of resolution. *P* values in bold are significant.

## Data Availability

The data that support the findings of this study are available from the corresponding author upon request.
